# Preoperative Heart Rate Variability and Parasympathetic Modulation in Newborns with Transposition of the Great Arteries Undergoing Arterial Switch Operation

**DOI:** 10.3390/children13081085

**Published:** 2026-08-15

**Authors:** Dilek Yavuzcan Öztürk, Demet Oğuz, Şenay Çoban, Erkut Öztürk

**Affiliations:** 1 Department of Neonatology, Istanbul Saglik Bilimleri University Basaksehir Cam and Sakura Hospital, 34003 Istanbul, Turkey; 2 Department of Pediatric Cardiology, Istanbul Saglik Bilimleri University Basaksehir Cam and Sakura Hospital, 34003 Istanbul, Turkey

**Keywords:** newborn, transposition of the great arteries, electrocardiography

## Abstract

**Highlights:**

**What are the main findings?**

- Heart rate variability progressively decreased before arterial switch surgery in neonates with transposition of the great arteries.
- High-frequency (HF) power was independently associated with increasing time to surgery, indicating worsening parasympathetic dysfunction.

**What is the implication of the main finding?**

- Heart rate variability monitoring may provide a non-invasive tool for detecting physiological deterioration before surgery.
- Early identification of autonomic dysfunction may improve preoperative monitoring and risk stratification in neonates with transposition of the great arteries.

**Abstract:**

Introduction: We retrospectively assessed preoperative cardiac autonomic regulation in neonates with TGA who subsequently underwent arterial switch operation. Materials and Methods: Neonates treated between 1 January and 31 December 2024 were studied. Heart rate variability was derived from continuous ECG recordings, with low-frequency and high-frequency spectral components calculated from RR intervals. Longitudinal associations between HRV indices and the interval to surgery were evaluated using linear mixed-effects models with adjustment for relevant clinical covariates. Results: Twenty neonates were included. The median age at ASO was 7 days (IQR 5–9), median weight was 3100 g (IQR 2900–3400), and median preoperative oxygen saturation was 76% (IQR 70–82%). Balloon atrial septostomy had been performed in 10 patients. HRV data represented 150 patient-days of monitoring. In unadjusted analyses, both LF and HF power declined as surgery was delayed, with the strongest association observed for HF power. In the adjusted mixed-effects model, longer time to surgery remained independently associated with lower HF power (*β* = −0.07, 95% CI −0.129 to −0.011, *p* = 0.03). Conclusions: Serial HF measurements may reflect progressive changes in autonomic regulation during the preoperative period in neonates with TGA.

## 1. Introduction

Transposition of the great arteries (TGA) is a major cyanotic congenital heart lesion encountered during the neonatal period and commonly requires early surgical correction. Because the systemic and pulmonary circulations run in parallel before repair, adequate mixing of oxygenated and deoxygenated blood is essential during the interval preceding arterial switch operation (ASO). Insufficient intracardiac or ductal mixing may result in severe hypoxemia, metabolic deterioration, and clinical instability, making close physiological surveillance particularly important [1,2,3,4].

Following birth, the circulatory system must rapidly adapt from fetal parallel flow to postnatal serial circulation. In neonates with TGA, this transition is especially vulnerable to disturbances in atrial, ventricular, or ductal mixing. Prostaglandin E1 is therefore frequently used to maintain ductal patency, while balloon atrial septostomy may be required when atrial communication is inadequate. These interventions support systemic oxygen delivery while the infant is awaiting definitive repair, but the preoperative period may still be accompanied by hypoxemia and hemodynamic stress [3,4].

Electrocardiography provides continuous information that can be obtained without additional invasive monitoring. Beat-to-beat variation in cardiac cycle length reflects the combined influence of sympathetic and parasympathetic activity and can be quantified as heart rate variability (HRV). HRV can be examined in both time and frequency domains, and changes in these measures have been associated with altered physiological states in critically ill neonates [5,6,7]. However, longitudinal preoperative HRV data in newborns with TGA remain limited. We therefore investigated whether HRV changes during the waiting period before ASO could provide information about evolving autonomic regulation.

The objective of this study was to characterize serial preoperative HRV in neonates with TGA undergoing ASO and to determine whether changes in HRV were associated with the time elapsed before surgical repair.

## 2. Materials and Methods

This study was conducted retrospectively between 1 January 2024, and 31 December 2024, in the pediatric cardiac intensive care unit of our hospital, on neonates diagnosed with transposition of the great arteries (TGA). Premature infants, patients diagnosed at over one month of age, cases with poor echocardiographic images, subaortic stenosis, pulmonary stenosis, arch hypoplasia or aortic coarctation, and those diagnosed with Taussig-Bing anomaly were excluded from the study. The study was conducted in accordance with the Declaration of Helsinki after obtaining local ethics committee approval.

All infants remained in the pediatric cardiac intensive care unit from admission until surgery and were managed according to the unit’s standard preoperative protocol with continuous ECG surveillance. HRV measurements were obtained under the same monitoring conditions and with the same ECG platform throughout the study. Midazolam and/or morphine were given only when clinically required rather than as routine sedation; eight infants (40%) received sedoanalgesia before surgery.

For each infant, demographic, clinical, medication, and echocardiographic information was recorded prospectively on a standardized study form. Medications were categorized as sedoanalgesic agents (fentanyl, morphine, or midazolam), vasoactive drugs used for hypotension (norepinephrine or epinephrine), neuromuscular blocking agents (vecuronium or rocuronium), and prostaglandin E1. Pain and sedation were evaluated with the Neonatal Pain, Agitation, and Sedation Scale (N-PASS) [8]. The scale incorporates behavioral and physiological observations, including irritability, behavioral state, facial expression, muscle tone, and vital signs. The target N-PASS range was −1 to +3. A vasoactive–inotropic score was calculated for infants who required vasoactive support for at least 24 h [9]. Low cardiac output was recorded when clinical evidence such as reduced urine output, elevated lactate, or impaired systemic perfusion was present.

Continuous ECG recordings were obtained from admission after birth until ASO using a Philips IntelliVue MX800 monitor (Philips Healthcare, Andover, MA, USA) at a sampling rate of 250 Hz. Clinical information was retrieved from the electronic medical record. The ECG signal was filtered between 0.5 and 70 Hz, and QRS complexes were detected automatically to generate sequential RR intervals (RRis).

For HRV assessment, RRi data were segmented into consecutive 10 min analysis epochs. The signal-processing software automatically screened the recordings for artifacts, ectopic complexes, and noisy RR intervals and corrected eligible abnormalities before calculation of HRV indices. Daily estimates were calculated as the mean of all quality-controlled 10 min epochs available for that day. Recordings that did not meet the software’s automated quality-control criteria were excluded from analysis; no additional minimum recording duration was imposed.

Frequency-domain measures were derived using the Welch periodogram. Each 10 min epoch was divided into consecutive 1 min windows, and the corresponding periodograms were averaged to obtain the spectral density estimate. LF power was defined over 0.05–0.25 Hz and HF power over 0.25–1.5 Hz. Values were logarithmically transformed and summarized as median spectral power. LF reflects a combination of sympathetic and parasympathetic influences, whereas HF is predominantly related to parasympathetic cardiac modulation. DFA-derived measures were also averaged as additional indices of HRV dynamics [5,10].

Time to surgery was defined as the number of postnatal days from birth to the day of the arterial switch operation.

### Statistical Analysis

Continuous variables were summarized as median (interquartile range [IQR]) and categorical variables as counts and percentages. Analyses were performed using IBM SPSS Statistics, Version 23 (Statistical Package for the Social Sciences for Windows).

Longitudinal changes in HRV and clinical variables were evaluated with linear mixed-effects models incorporating subject-specific random intercepts and slopes. For infants receiving vasoactive therapy, the highest daily vasoactive–inotropic score was used. Low cardiac output status and the daily maximum vasoactive–inotropic score were treated as time-varying covariates. Potential collinearity was examined using the variance inflation factor, with variables showing VIF > 10 excluded. Residual behavior and model fit were checked during model development, and no important violations of the model assumptions were identified. Because both LF and HF were related to time to surgery in the unadjusted analyses, an adjusted model was constructed. Given the limited cohort size, HF was selected as the primary outcome because it showed the strongest unadjusted association and has a closer physiological relationship with parasympathetic modulation. Statistical significance was defined as *p* < 0.05.

## 3. Results

Twenty neonates with TGA who underwent ASO were included; 12 (60%) were male. Median age at surgery was 7 days (IQR 5–9), median body weight was 3100 g (IQR 2900–3400), and median preoperative oxygen saturation was 76% (IQR 70–82%). Balloon atrial septostomy was performed in 10 infants. Baseline clinical and anatomical characteristics are summarized in Table 1.

Across 150 patient-days of ECG-derived HRV recordings, both LF and HF power showed an inverse relationship with the interval to surgery in the unadjusted analyses (LF estimate −3.4 × 10^−3^, *p* = 0.04; HF estimate −5.1 × 10^−3^, *p* < 0.001). HF demonstrated the strongest association. After adjustment for the prespecified clinical covariates, each additional day before surgery remained associated with a reduction in HF power (*β* = −0.07, 95% CI −0.129 to −0.011, *p* = 0.03).

The adjusted model therefore identified time to surgery as the only statistically significant factor associated with HF power. The complete model estimates and confidence intervals are presented in Table 2.

## 4. Discussion

In this cohort of neonates with TGA awaiting ASO, serial HRV assessment showed a measurable reduction in HF power as the interval to surgery lengthened. This association persisted after accounting for vasoactive support, low cardiac output, birth-weight z-score, sedation/pain score, and mechanical ventilation. The findings suggest that autonomic regulation may change during the preoperative course and that HF power could provide an additional physiological signal alongside conventional clinical monitoring.

The period between birth and definitive repair is physiologically demanding for infants with TGA. The adequacy of circulatory mixing, ductal patency, atrial communication, respiratory support, vasoactive therapy, and the presence of low cardiac output can all influence the infant’s condition. Autonomic regulation may represent another component of this complex adaptive response [11]. Previous work in neonates with critical congenital heart disease has reported reductions in HRV during the preoperative period [5]. Our results extend this observation to a focused TGA population by demonstrating a longitudinal relationship between HF power and time to ASO.

HRV-based monitoring has been explored in several neonatal conditions. Sullivan et al. [12] reported associations between abnormal heart rate characteristics and clinical conditions such as infection, inflammation, respiratory failure, and surgery in very-low-birth-weight infants. In another neonatal cohort, changes in HRV preceded the clinical diagnosis of necrotizing enterocolitis, suggesting that autonomic measures can change before overt clinical deterioration [13]. Presacco et al. [14] likewise showed that HRV-derived RMSSD was associated with neurological injury severity in neonates with hypoxic–ischemic encephalopathy undergoing therapeutic hypothermia. Together, these studies support the potential value of longitudinal HRV measurements as complementary physiological markers, although the underlying mechanisms and clinical implications differ between disease states.

Al-Shargabi et al. [13] examined HRV changes surrounding necrotizing enterocolitis and found evidence of altered autonomic tone before clinical diagnosis. Their findings indicate that the temporal behavior of HRV may contain information about evolving illness severity. In our study, the direction of change was also longitudinal, but the clinical setting and endpoint were different: we evaluated neonates with TGA during the period leading to planned cardiac surgery rather than before an acute inflammatory diagnosis.

Presacco et al. [14] used continuous HRV monitoring in neonates with moderate-to-severe hypoxic–ischemic encephalopathy treated with therapeutic hypothermia and found that RMSSD was related to subsequent brain injury severity. Although our study used frequency-domain measures rather than RMSSD, both observations illustrate that changes in cardiac autonomic signals may accompany important physiological stress in critically ill newborns.

Evidence specific to congenital heart disease remains comparatively limited. Siddiqui et al. [15] reported differences in fetal autonomic regulation among fetuses with congenital heart disease, including HLHS, TGA, and TOF, compared with controls. Mulkey et al. [11] also described depressed HRV during the early postnatal transition in infants with complex congenital heart disease. These observations are consistent with the possibility that autonomic imbalance begins early in the neonatal course and may be influenced by the severity of circulatory adaptation.

Our findings are also broadly consistent with the longitudinal observations of Govindan et al. [5], who studied neonates with critical congenital heart disease, including TGA and HLHS, before surgery. Their study reported declining HRV over the preoperative period, whereas our analysis specifically demonstrated an independent association between longer time to ASO and lower HF power in TGA. The agreement between these findings supports the reproducibility of a longitudinal HRV signal, while the disease-specific design of our cohort provides a more focused assessment of the pre-ASO period.

HF power is commonly used as a frequency-domain measure that predominantly reflects vagal cardiac modulation. In our cohort, the decline in HF with increasing time before surgery may therefore represent reduced parasympathetic influence. This interpretation should remain cautious because HF is affected by respiratory pattern, developmental maturation, medications, and signal-processing methods [16,17]. Accordingly, the observed association should be viewed as a physiological marker rather than direct proof of isolated parasympathetic dysfunction.

Recent work has further highlighted the value of HRV in infants with TGA. Somasundaram et al. [17] found that pre-discharge HRV assessment was informative for autonomic regulation, although a single measurement was not independently related to later neurodevelopmental outcomes. This supports the concept that repeated measurements may be more informative than a single HRV snapshot. In the present study, serial HF assessment captured changes during the interval before ASO and may therefore have greater potential for monitoring than a one-time measurement.

## 5. Limitations

Several limitations should be considered. First, this was a retrospective, single-center study with only 20 infants, which limits statistical power and increases the possibility of overfitting. To reduce model complexity, HF was chosen as the primary adjusted outcome based on its strongest unadjusted association. Second, some ECG recordings were incomplete or unavailable because of technical limitations or the clinical instability of individual infants. Third, not every clinical covariate was available for every recording day. Although the mixed-effects approach permits longitudinal analysis with incomplete observations under its modeling assumptions, residual bias from missing information cannot be excluded. Finally, treatment before surgery was individualized. Vasoactive medications, mechanical ventilation, and sedoanalgesia may alter autonomic measurements and could confound the observed associations. These limitations warrant confirmation in larger prospective multicenter cohorts.

## 6. Conclusions

Among neonates with TGA awaiting ASO, HF power decreased as the preoperative interval increased, and this relationship remained significant after adjustment for selected clinical factors. Serial HRV assessment may therefore offer complementary information about changing physiological status before surgery. Prospective multicenter studies with larger samples are needed to determine whether these HRV changes have prognostic value and whether integrating HRV with established clinical variables improves preoperative risk assessment.

## Figures and Tables

**Table 1:** General characteristics of the patients.

Variables	
*n*	20
Weight, kg, median (IQR)	3.1 (2.9–3.4)
Male, *n* (%)	12 (60)
Syndrome, *n* (%)	1 (5)
Prostaglandin E1	16 (80)
Inotrope	3 (15)
Neuromuscular blocker agents, *n* (%)	2 (10)
Sedoanalgesia, *n* (%)	8 (40)
N-PASS, median (IQR)	2 (0–3)
Mechanical ventilation, *n* (%)	2 (10)
Preoperative saturation, median (IQR)	76 (70–82)
Age at surgery/day, median (IQR)	7 (5–9)
Balloon atrial septostomy, *n* (%)	10 (50)
Usual coronary artery, *n* (%)	12 (60)
Unusual coronary artery	8 (40)
1LAD2 RCx (1 intramural)	4 (20)
1LCxR	1 (5)
2RLCx (1 intramural)	1 (5)
1LRCA 2Cx	1 (5)
1R 2LCx	1 (5)
Aortic–pulmonary artery relationship, *n* (%)	
Anterior and to the right	14 (70)
Side-by-side, aorta to right	3 (15)
Directly anterior	1 (5)
Posterior and to the right	1 (5)
Anterior and to the left	1 (5)
Ventricular septal defect, *n* (%)	5 (25)
Atrial septal defect (nonrestrictive), *n* (%)	6 (30)
Patent ductus arteriosus, *n* (%)	18 (90)

Cx: Circumflex; LAD: left anterior descending; PDA: patent ductus arteriosus; N-PASS: The Neonatal Pain, Agitation, and Sedation Scale, PGE1; RCA: right coronary artery. Sedoanalgesia consisted of midazolam and/or morphine administered when clinically indicated.

**Table 2:** Results of the linear mixed-effects model examining factors associated with heart rate variability (HF).

Variable	Estimate (*β*)	Standard Error	95% Confidence Interval	t-Value	*p*
Time	−0.07	0.03	−0.129 to −0.011	−2.33	0.030
Vasoactive inotropic score	0.24	0.12	−0.009 to 0.475	2.00	0.06
Low cardiac output state	−0.12	0.22	−0.551 to 0.311	−0.55	0.58
Birth-weight z-score	−0.20	0.25	−0.690 to 0.290	−0.80	0.42
The Neonatal Pain, Agitation, and Sedation Scale	−0.42	0.45	−1.302 to 0.462	−0.93	0.35
Mechanical ventilation	−0.18	0.15	−0.474 to 0.114	−1.20	0.23

## Data Availability

The data supporting the findings of this study are available from the corresponding author upon reasonable request. The dataset is not publicly available because it contains patient-level clinical information subject to privacy and ethical restrictions.
